# Community perceptions of enrolment of indigents into the National Health Insurance Scheme in Ghana: a case study of the Livelihood Empowerment against Poverty Programme

**DOI:** 10.1186/s41256-022-00238-2

**Published:** 2022-01-28

**Authors:** Patricia Akweongo, Edmund Voetagbe, Fabrizio Tediosi, Dominic Dormenyo Gadeka, Paola Salari, Moses Aikins

**Affiliations:** 1 School of Public Health, University of Ghana, Accra, Ghana; 2 Swiss Tropical and Public Health Institute (Swiss TPH), Socintrasse 57, 4051 Basel, Switzerland; 3 Institute of Pharmaceutical Medicine (ECPM), University of Basel, Basel, Switzerland

**Keywords:** National Health Insurance Scheme, Ghana, Livelihood Empowerment against Poverty, Community perception, Community-based approach

## Abstract

**Background:**

The Livelihood Empowerment against Poverty (LEAP) programme in Ghana as part of its beneficiary programme, identifies the poor/indigents for exemptions from premium payments in the National Health Insurance Scheme (NHIS). This paper sought to understand community perceptions of enrolling the poor in the NHIS through LEAP in order to inform policy.

**Methods:**

The study adopted a descriptive cross-sectional study design by using a qualitative approach. The study was conducted in three geographical regions of Ghana: Greater Accra, Brong-Ahafo and Northern region representing the three ecological zones of Ghana between October 2017 and February 2018. The study population included community members, health workers, NHIS staff and social welfare officers/social development officers. Eighty-one in-depth interviews and 23 Focus Group Discussions were conducted across the three regions. Data were analysed thematically and verbatim quotes from participants were used to support the views of participants.

**Results:**

The study shows that participants were aware of the existence of LEAP and its benefits. There was, however, a general belief that the process of LEAP had been politicized and therefore favours only people who were sympathizers of the ruling government as they got enrolled into the NHIS. Participants held the view that the process of selecting beneficiaries lacked transparency, thus, they were not satisfied with the selection process. However, the study shows the ability of the community to identify the poor. The study reports varying concepts of poverty and its identification across the three ecological zones of Ghana.

**Conclusion:**

There is a general perception of politicization and lack of transparency of the selection of the poor into the NHIS through the LEAP programme in Ghana. Community-based approaches in the selection of the indigent are recommended to safeguard the NHIS-LEAP beneficiary process.

## Background

In the implementation of social health insurance, the need to protect the poor from paying premiums has led to several initiatives on how to identify and target the poor for exemptions. The concept of poverty is noted to be complex and multi-dimensional and hence does not apply to only the absence of money but includes food security, access to basic health services, socioeconomic status, living conditions and possessions of assets [1]. Getting access to health services is one of the most difficult aspects of life especially, for the poor. In low- and middle-income countries, the most poor often lack access to health services [2]. The connection between poverty and access to health services can be seen as part of a higher phase, where poverty leads to ill health and ill health retains poverty [3]. The most poor in the population are often weakly represented in public policy making. Public policy decisions are often taken behind closed doors, as technocratic solutions with limited or no consultation, potentially resulting in lack of public ownership, and weak accountability [4]. Nationwide multilateral discourse, with government, employers and workers as well as academics, civil society and others, is fundamental to adopting optimal public policies. Public discourse is required to guarantee social protection systems and a socially responsible retrieval expected at attaining comprehensive development and social justice [4].

In a context of widespread poverty, determining who really is poor can be challenging. Several processes have been used to identify the poor including administrative processes, community processes and mixed method approaches. Administrative processes involve a higher authority such as the ministry or government deciding on who they define as poor. Thirteen percent of indicators used to assess poverty are often derived from household surveys [1]. With the introduction of the National Health Insurance Scheme (NHIS), Ghana has been using the means test and, more recently, the eligibility to the Livelihood Empowerment against Poverty (LEAP) to identify the beneficiaries as poor for exemptions from premium payments. LEAP is a social cash transfer programme, established in 2008 and funds are disbursed bi-monthly to beneficiaries. The programme is funded from revenues of the Government of Ghana (50%), donations from the Department for International Development (DFID) and a loan from the World Bank, which aims to provide cash and health insurance to extremely poor households across Ghana in order to alleviate short-term poverty and encourage long-term human capital development [5]. The beneficiaries of the LEAP, however, must adhere to some conditions such as sending their children to school and avoiding child labour.

All LEAP beneficiaries are also entitled to free registration with NHIS. The benefit package of the NHIS covers over 95% of all diseases that affect the Ghanaian population [6]. It covers out-patient services and most-inpatient services (specialist care, most surgeries, cervical and breast cancer, physiotherapy, hospital ward accommodation), oral health, eye services etc. It provides free maternal health care to all pregnant women and post-natal care up to 90 days. It also covers Caesarean delivery, emergency care and all drugs in the NHIA (National Health Insurance Authority) Medicines lists and traditional medicines approved by the Ghana Food and Drugs Authority [7].

Eligibility for LEAP is based on poverty status and having at least one of the three demographic conditions such as households with orphan or vulnerable children (OVC), elderly poor and persons with extreme disability [8]. Initial selection of poor households is done through a community-based process and is verified centrally with a proxy means test. Within the category of extreme poor, the programme further targets households with one or more (a) persons who are over 65 years of age, (b) persons living with a severe disability, and (c) caregivers of orphans and vulnerable children. However, since 2016, as a result of the new initiative known as LEAP 1000, the programme has also targeted households with a pregnant woman and children below 1 year of age [9].

Observation of the LEAP approach to identifying the poor and vulnerable shows a mixed method approach where a national poverty map generated through the Ghana Living Standards Survey is first used to rank the regions and districts. Regions and districts selected depend on their location on the poverty map. Subsequently, the communities selected depend on their poverty ranking at the district level [10]. Hanson et al. [11] described six different mechanisms that have been applied in the health sector, including resource allocation formulae, contracting NGOs, user fee exemptions, cash transfers, vouchers, and market segmentation strategies. The LEAP applies the means test of cash transfers. However, the approach to identifying the poor, means of verification of the ‘real poor’ and how much it is able to reduce the leakage of the true poor into the wider premium group needs to be evaluated. Inability to identify the real poor could lead to high health care expenditure with implication for sustainability of the NHIS. Additionally, community perception of this criteria for identifying the poor and enrolling the poor/indigents into the NHIS through LEAP has not been established. This paper sought to understand community perceptions of using the LEAP programme in enrolling indigents into the NHIS in Ghana. In addition, the study explored reasons for low NHIS enrolments and low renewals.

## Methods

### Study design

The study adopted a descriptive cross-sectional design by using community-based qualitative approach to understand community perceptions of enrolling indigents into the NHIS through LEAP. Eighty-one IDIs and 23 FGDs were conducted with study participants. The study was conducted between October 2017 and February 2018 in three geographical regions of Ghana. The qualitative method was used to solicit participants’ views in more detail about the enrolment of indigents. This has contributed to a better understanding of the criteria used to identify the poor for enrolment into the National Health Insurance Scheme of Ghana and reasons for low NHIS enrolment as well as low renewals.

### Study setting

This study was conducted in three out of the 10 administrative regions of Ghana, namely, Greater Accra, Brong-Ahafo and Northern regions. These regions represent Ghana’s three ecological zones (southern, middle and northern zones). The regions are characterized by mixed urban and rural populations. The Greater Accra Region is a coastal region with 4.94 million inhabitants and is the region with the lowest poverty incidence in the southern belt with poverty head count of 5.6%. Brong-Ahafo region is located within the middle belt with 2.85 million inhabitants and the region with the high poverty incidence in the middle belt with poverty head count of 27.9%, while the Northern Region represents the northern belt with a population of 3.06 million and with the highest poverty incidence in this belt with poverty head count of 50.4% [12, 13]. Two districts with relatively high enrolment of indigents from the 2015 membership data of the NHIS were purposively selected from each of three study regions. These districts also have all the level of health facilities: Community-Based Health Planning and Services (CHPS), Health centers, Hospitals, Clinics, Laboratories, Chemical and Pharmacy shops. Additionally, the districts have well established District Health Insurance Scheme offices and the proportion of indigents within these districts ranged from 4 to 20% [14].

### Study population and sampling

The study population included community members, health workers, NHIS staff and social welfare/social development officers in the selected districts of the three regions. The majority of participants were community members comprising of leaders and poor indigenous people. The participants were selected by using stratified purposive sampling method. This involved the selection of participants across relevant groups dealing with the same phenomenon where each group was homogenous enough so that cross-group comparisons could be made [15]. According to deMarrais [16], face-to-face interviews provide perspectives and insights into special knowledge that only participants possess. Generally, information gathered included community definition and identification of the poor in community, registration renewal, use of NHIS for health services and perceptions of enrolling indigents in the NHIS through the LEAP.

### Data collection

Data were collected by using In-Depth Interviews (IDIs) and Focus Group Discussions (FGDs). These qualitative methods were employed because they produce very rich source of data which reveal the world of the participants, their emotions and thoughts about the world around them [14]. This approach was also to offer the participants an atmosphere that encourages them to tell their own stories regarding the issues of LEAP and NHIS without interference. It was to provide them the flexibility, which in turn, allowed the investigator to seek further clarification.

In all, 23 FGDs and 81 IDIs were conducted across the three regions. The IDIs were conducted among various categories of health workers including nurses, midwives, physician assistants and health administrators, LEAP officers and NHIS officers. In addition, some of the interviews were held with those enrolled as indigents under the NHIS as well as the poor identified by the communities that were not enrolled. The FGDs were conducted with community members. Participants shared views on their understanding and perceptions of poverty, criteria for identifying the poor and perceptions of enrolling indigents into the NHIS through the LEAP.

The study objectives and scope were explained to all participants prior to their participation in the interviews. Confidentiality was assured and with permission from participants, the interviews were recorded. Manual note-taking was also carried out to complement the recording and to serve as a backup. Both the recording and the notes helped to ensure accuracy of data and also facilitated data analysis processes. Reflective interview guides containing open ended questions were used in the study. Participants were interviewed in local languages spoken in the three study regions. However, some of the participants who were comfortable and could speak English fluently opted to be interviewed in English.

In order to minimize respondent bias and the risk of reactivity, the questions were asked by following the cues from the participants. Appointments were booked with potential participants before the interviews were conducted. All interviews and discussions took place where privacy was assured and at the convenience of the participants. Interviews and discussions lasted between 30 min and 2 h.

### Data analysis

Information gathered during the data collection was manually analysed. Firstly, the conversation notes were developed into field note books at the end of each day and recorded interviews were transcribed verbatim in Microsoft word. Verbatim transcription is the word-for-word reproduction of verbal data, where the written words are an exact replication of the audio-recorded words [17]. Participant validation was carried out to ensure trustworthiness and this was done by debriefing the analytical results with participants for agreement [18]. This was possible because initial transcription was done immediately after the interview. Some of the transcribed data were transcribed and translated from a local language into English. The transcript and notes were read several times to get a sense of the entire data. Secondly, the transcript was subjected to a qualitative content analysis to generate common themes emanating from the data: definition and identification of the poor in community, registration renewal, use of NHIS for health services and perceptions of enrolling indigents in the NHIS through the LEAP programme. Thirdly, typical comments by the categories of participants: community members, health workers, NHIS staff and social welfare officers/social development officers were abridged into meaningful summary statements and further condensed to form themes. The authors then critically reviewed the transcription and the analysis to discuss the entire results to ensure that the themes and summary statements reflect participants’ views. Statements of the respondents were presented as quotes to substantiate the views expressed.

## Results

### Identification of the poor for enrolment into the National Health Insurance Scheme

The qualitative interviews gathered from different stakeholders (Table 1) shared light on community perceptions of poverty and the enrolment processes in the community.

**Table 1:** Background information of participants

Participants information	Greater Accra	Brong	Ahafo	Northern (NR)	Total
	(GAR)	(BA)			
Data collection strategy					
FGDs	7	8		8	23
IDIs	14	32		35	81
FGDs number/sex					
Males	4	4	4		12
Females	3	4	4		11
Number of participants in FGDs					
Males	38	40		40	118
Females	25	39		38	102
IDIs sex					
Males	8	16		23	47
Females	6	16		12	34
Type of participants					
Community members	13	21		17	51
Health workers	0	6		10	16
NHIS staff	1	2		4	7
Social welfare officer/development officer	0	3		3	6

The views expressed by participants suggested that there was no consensus on the definition of a poor person in the community. However, the majority defined poverty as the inability of a person to afford basic necessities of daily living. These activities of daily living included food, clothing, and payment for health care and school fees of children. Therefore, individuals in the community who had challenges in meeting these needs in the community were classified as being poor. The following quotes illustrate these points:

Poverty is when you farm and you are not able to get a good yield, feeding your family becomes a problem; you are unable to pay school fees, this is what we term as poverty (Male, FGD, NR).

The poor are persons who are not able to feed themselves and their children. They cannot pay the school fees of their children as well as hospital bills when they are sick. We have a lot of poor people in the villages (female, FGD, BA).

Both FGD and IDI participants across the three study regions identified orphans, elderly, people who are sick and unable to work and people with disability challenges as poor people in the community. Participants also characterized widows as poor because they did not have husbands to take care of them. They observed that widows with children often found it difficult to cater for these children especially when they were unemployed.

In this community we know each other very well; and we know the less privilege in this community, such as physically challenged, widows/widowers and orphans when such programme come into the community, we encourage them to go and register so that they can benefit from such programmes (Male, FGD, BA).

The poor in the community are the orphans, people who are sick and cannot work and women whose husbands have died leaving them with children to take care of. They suffer a lot as they are not able to feed the children, take care of their fees and the children will always appear in dirty and torn clothes (Female, FGD, NR). We have orphans, people with disability and those who are sick and cannot work are the poor people in this community. They need help because they have nobody to take care of them (Male, IDI, GAR).

There were little variations in the definition of the poor across Brong Ahafo and Northern regions. Table 2 provides a summary of participants’ definition and people identified as poor in the community across the three study sites.

**Table 2:** Participants’ definition and people identified as poor across the three study regions

Region	Definition of poor	Identification of the poor
Brong Ahafo	Inability to feed self and family Cannot send children to school and pay school fees Sick person who cannot work or far Unable to renew insurance Cannot pay Hospital bill Widow Aged without a helper Inability to afford good clothing	Orphan People with disability Dressing of their children Physical appearance Type of house the person lives in
Northern region	Inability to feed self and family Cannot send children to school and pay school fees Sick person who cannot work or far Unable to renew insurance Cannot pay Hospital bill Widow Aged without a helper Inability to afford good clothing	Orphans Disability Children wearing tattered clothes Appearance and dressing Live in dejected houses without light
Greater Accra region	Sick person who cannot work	Orphans People with disability Dressing of the person and family members Physical appearance (tattered and dirty clothes)

### Identification of the poor in the community

In both IDI and FGD, participants were of the view that it was easy to identify poor people in the community. The poor people could be identified through their general appearance, type of house the person lives in, type of food they eat as well as their inability to pay children school fees and hospital bills. Some participants reported that poor people often lived in houses with no electricity because they could not afford to pay light bills, they or their children were often seen in tattered clothes and they were often withdrawn from the community. Participants perceived that people who lived in the community with the poor for instance, community leaders could be employed to identify the poor.

Identifying poor people in this community is easy. We know them from the place they live, clothes they wear and the way they even talk and carry themselves around. They are often quiet and do not participate in community activities (Male, IDI, BA).

It is not easy to identify the poor, you can only identify the poor when the person is sick and can’t afford medical bills, until someone comes to his/her aid, such a person will not be able to pay. Again the physically challenge cannot work and cater for themselves, they live on receiving alms and live in houses without light because they cannot afford to pay the bill (Male, FGD, NR).

### Community structures for identifying the poor

In both IDIs and FGDs, community members purported to know the people because they lived with them and also had fair understanding of their situation. The community leaders including religious leaders were people that could be employed to identify the poor in addition to house-to house identification. In their view, religious leaders could easily identify poor members among their congregation. Besides, poor people could be identified by health workers who attend to patients at the health facility level. This was because poor people are often not in the position to pay for health care services they received. Both community members and health workers were of the view that the hospital could be used to recruit the poor. These suggestions are supported by the following quotes:

Community leaders could be employed to identify poor people. The church leaders know the people who are poor in their church. So, if you want to identify poor people in the community, they should be contacted to assist (Female, IDI, GAR).

We have leaders in this community, so they can be tasked to identify these people who are poor because they know them and after that the government should send someone, for instance, a CID (Criminal Investigating Department) officer to confirm that the people selected are really poor. The reason is that some people would be left out if you just ask just everybody to do the selection. This could lead to selecting those who are not poor tobenefit from the support (Male, IDI, GAR).

The assembly men and women in the sub-districts know their people and they are in contact with them; even though we have community health nurses who go for home visits and outreaches, they could reach out to them but those will be few. An assembly man is from the community and stays with them; he is, therefore, in a better position to identify poor people. So, we could contact the assembly men to help identify the poor and register them for free (36 years midwife, IDI, BA).

…the hospital is one of the places you can use to identify the poor because they cannot afford to pay the hospital bill after treatment. They cannot even buy food when they have been admitted in the hospital (Female, FGD, NR).

Table 3 presents a summary of community strategies participants identified as means of identifying the poor across the three study sites.

**Table 3:** Community strategies for identifying the poor proposed by respondents

Region	Strategies to identify the poor
Brong Ahafo	Employment of community leaders (chiefs and assemble members Employment of religious leaders House to house identification Employment of social welfare officers Use of health facilities (attendees who are unable to pay for care they received)
Greater Accra region	Community leaders (chiefs and assembly members) Employment of religious leaders
Northern region	Employment of community leaders Employment of religious leaders House to house identification Employment of social welfare officers Attendees who are unable to pay for the care they have received

### Reasons for low NHIS enrolment and low renewals

IDI and FGD participants acknowledged the importance of the NHIS in increasing access to health care. However, some participants did not renew their membership with NHIS because members were given poor quality healthcare services when they visited health care facilities. Others maintained that they still needed to pay for services even though they had active cards and that discouraged them from renewing their NHIS cards.

What I have to say is that the reason why people are not enrolling is that when you use the NHIS card, what you get like drugs is paracetamol. Sometimes, even paracetamol which cost 1 cedi 50 pesewas (US$0.20) you will be told to pay and it’s not covered (Male, FGD,GAR).

When you have the card and go to hospital, you will be told that this drug is not there or it is not covered by the insurance. So, many of us do not register or renew the card because of that. If they want people to register or renew their card, it should cover all medicines and sicknesses (Female, FGD, NR).

Negative attitudes from health workers and discrimination against NHIS card holders also affected renewal and enrollment into NHIS according to views shared by participants. One participant put it as*:*

There is a discrimination between those who have the insurance and those who don’t have the insurance. Those with insurance are packed aside and then priority is given to those who have money to pay. The doctors do not even pay attention to you when you go there with a condition. A child can be rushed to the hospital with high temperature but they won’t even bother to attend to the child. So, if you want better health care and proper medicine, you had better go without the card (Male, FGD, BA)

Despite this, health workers who participated in this study did not agree that they provided differential services to insured and non-insured clients. According to them, all clients went through the normal procedure in the health facility and were provided with client-specific health care.

When the person comes and he/she is an insured client, he/she goes through the normal procedure and goes away without any charge except that the person needs some drugs that we don’t have at our health facility and we write for the person to go and buy outside. Other than that, if the person is having the insurance and we have everything, then the person will go through the process from beginning till he/she goes away without any further charge. But, for the non-insured, they also go through the normal process but they are charged to pay before taking their drugs and go (Midwife, BA)

Another reason for non-renewal was the challenges involved in the process. Apart from the renewal centres located far away from the community, participants complained about the delays in the processes. To get a card renewed could require spending several hours at registration centres and in some instance, one would have to go to renewal centres several times before being served. The following quotes support these points:

I am registered because with the card you get free treatment at the hospital when you are sick. If you are poor and you don’t register then you will struggle to pay the hospital bills. So, the insurance is good for everybody especially the poor, but renewal is the problem because you have to travel far to the centre for renewal. The last time, I had to go there three times before they could renew my card (Male, IDI, NR).

### Livelihood Empowerment against Poverty (LEAP) for enrolling the poor

Participants were aware of the existence of LEAP programme in the community. They perceived that LEAP was a useful intervention in reducing poverty in the country as demonstrated in the following quotes from the interviews:

The LEAP money helps us a lot because I have two children in school and I use some of the money to pay for their fees and use the rest to care for the family. I have used the rest of the money to buy fowls so that when I am out of money, I could sell one and use the money for something (Female, IDI, BA).

We have LEAP in this community and it is very useful. I know people who are benefiting from it and it is helping them a lot (Female, FGD, NR)

However, there was a general belief that the program has been politicized and only people who are sympathizers of the ruling government were selected to benefit from the programme. This was reported in 15 FGDs of male and females and in 30 of the in-depth interviews. In a response to a question on knowledge about LEAP in the community, a participant of the IDIs in the Northern region had this to say in the quote below:

There is LEAP in this community but it has been politicized. When they came, they only sent it to communities that have party faithful and they are benefiting from it. It was when they told an assemblyman that the LEAP team would be coming to pay beneficiaries in one town that I got to know about the LEAP programme and the beneficiaries here. It angered other community members that they are not benefiting from the LEAP programme but I explained that it will get to their turn (IDI AS.)

In the FGDs in Brong Ahafo region, participants generally agreed that LEAP had been politicize and many beneficiaries were actually not poor people. Participants advocated for the employment of chiefs to distribute such welfare schemes for the community in the future as illustrated in the quote below:

Like my brother said if there is such help, it should not be passed through the politician but the chiefs who will get opinion leaders to assist them to identify and distribute the money. This will help but our politicians look for their political interest and select their party members. So, it should be through the chiefs who will select opinion leaders to help identity the poor (Male, FGD).

The selection of beneficiary districts and communities was often based on the criteria which included the level of poverty in the community by using the Ghana poverty map, access to health care and schools in the community:

In fact, doing the selection here, a team from Accra met with the municipal implementation team and the criteria they considered were for communities that are most vulnerable, then accessibility to health facilities and accessibility of schools and other social amenities and those were the criteria used in selecting the communities (Social Development Officer, IDI, BA).

People who were above 65 years without any help, orphans, people with disability and pregnant women with children under 1 year were reported as automatic beneficiaries of LEAP. Selected beneficiaries were verified in the community before disbursements were made to them.

In fact, the categories are the aged who are 65 years and above without any support and the severely disabled without means of livelihood and orphans and vulnerable children, pregnant woman and those with children under 1 year. These are the criteria for selection on to the LEAP (Social Worker, IDI, BA).

….We collect information from collateral source before we get to the homes to elicit from them whether actually they are poor. When you pay a visit to a place, you ask collateral sources around, and then you go into the household, you will see some of the characteristics. Then, we also try to look at our criteria, fit it in, and see whether he/she merits it. We don’t just pick people; we have to go through certain criteria. We need to go through certain things to justify that this man, when they say he is poor, he is poor (Social Development Officer, NR).

Nonetheless, participants were of the view that the process of selecting beneficiaries was not transparent as it is deemed to be politicized. Hence, only people who had political inclination to the incumbent government were selected. According to the participants, this process created a situation where many poor people in the community were left out, whilst others who, in their opinion, were not qualified to benefit were selected.

We were asked to come and write our names and the government was going to support the poor and needy, but when the support came, we the poor and needy were not added to the list. You see people who are strong and healthy are enjoying the package. I know a woman whose husband had died and no help coming from anyone but she doesn’t get the government package. There is an assemblywoman who is beneficiary of LEAP. She is strong and working (Female, FGD, BA).

The process is not transparent at all. In my community, they came and took the names and when they came back to distribute the money, some names were dropped from the list. We have people who live in this community and are very poor and yet they were not given anything. But others who are better off were given the money (Male, FGD, NR).

Truly, when the registration for LEAP was on going, they told us they were registering old and poor people. Two old women in my household registered including some young ladies from other households. But when the date was due for them to come for their monthly money the names of the two old ladies were omitted, while the young ladies had theirs (Male, FGD, GAR).

Some assembly members who were interviewed also alluded to the fact that the criteria were not transparent and coupled with the fact that community leaders were not involved in the selection process. An assemblyman in the Brong Ahafo Region had this to say in the quote below:

Yes, we have some of the LEAP in this community. When it came, we heard the information and we gathered the people, but the category for selection is not known to any assembly member and even when it’s time to pay them, no assembly member or committee member is in the known. So, we don’t even know the schedule of payment and the time they come, either do we know how much they are given (Assemblyman, IDI, BA)

Some participants suggested that involving more community leaders could improve the process of selecting beneficiaries and could make the process more transparent. Also, community sensitization on the process and selection criteria could help disabuse the belief about the politicization of the process and enhance transparency.

The public should know and be informed about the activities and processes. The chiefs and opinion leaders should be informed, so they could get the whole community members informed. Once the announcement is done, it will make everyone aware of the process and all the people involved can benefit from the process, so that it will not be given to just some selected few in the community (Male, IDI, BA).

There is need to educate community members about the process of selecting the people and involve the chiefs and opinion leaders. In doing that, people will see the process more transparent than what they do now. Just coming to write names and returning with few names as beneficiaries make people think it is given to only party members (Male, FGD, NR).

We do partner with the social welfare and then enroll the poor onto the scheme. Social welfare has identified these poor people through the LEAP and we are also able to enroll them free of charge into NHIS (Male NHIS staff, IDI, NR).

## Discussion

This paper sought to understand community perceptions of using the LEAP programme in enrolling indigents into the NHIS in Ghana. The study findings show that participants are aware of the existence of LEAP and its benefits. The study, however, reveals some negative perceptions of enrolling indigents into the NHIS through the LEAP. There was a general belief that the programme has been politicized and therefore favours only people who are sympathizers of the ruling government. Participants also noted that the process of selecting beneficiaries lacks transparency. As a result, communities are not satisfied with the entire selection process. These findings are consistent with a study on the success of interventions targeted at the poor where errors of inclusion and exclusion are often a result of efforts to “vote catch” by politicians and plain rent-seeking [19]. An earlier study by Tesliuc [20] highlighted the advantages and disadvantages of using local actors to select the poor. Whereas it generates accurate information on who to select as the poor may also generate conflict. Our findings confirm earlier studies where despite the improvement in the exemption criteria of NHIS and the general increase in the awareness of LEAP, the LEAP does not adequately resolve the problem of excluding the poor from the NHIS [21, 22]. Additionally, it raises more questions as to who qualifies to be exempted and who is not under the NHIS. It is therefore not surprising that an earlier study reported that about 30% of the 65% of the NHIS members who were exempted from paying premiums could indeed afford to pay [23]. Moreover, our study shows a lack of compliance with the conditions under the LEAP programme. However, this could be because the beneficiaries and potential beneficiaries are not aware of the sanctions regarding non-compliance. An earlier study with similar findings on lack of transparency and political interference, reported that those perceived biases could possibly be as a result of errors that resulted from practical difficulties in ensuring effective targeting within such rural contexts and inaccurate data on household poverty status within the informal sectors of rural communities [24]. Based on the broad coverage of our study in three ecological zones of Ghana, our findings indicate that the use of community structures could reduce the practical difficulties in targeting the poor, as the eligibility for LEAP is based on poverty status and having a household member in at least one of three demographic categories: households with orphan or vulnerable children (OVC), elderly poor, or persons with extreme disability unable to work. The participants were therefore of the view that involving more community leaders could improve the process of selecting beneficiaries and also make the process more transparent.

Our study shows that there existed no consensus on definition of who the poor are. However, the majority defined poverty in terms of ability to afford basic necessities of daily living. The criteria used to identify the poor included people who are unable to feed themselves and their family members, people who cannot pay their children school fees, sick persons who cannot work, the elderly people without a helper,, orphans, people with disability and the poor quality of house the persons live in. This confirms that the concept of poverty is complex and multi-dimensional and does not apply to only the absence of money but includes food security, access to basic health services, socioeconomic status, living conditions and possessions and assets as noted in an earlier study [1]. Moreover, these various concepts of poverty varied across the three ecological zones or regions. This clearly shows that poverty is a context-specific phenomenon [25]. However, the community has a clear understanding of who the poor are and moreover, the identification of the poor should be context specific. Additionally, this suggests that the community would be in a better position to identify the core poor under the LEAP to be enrolled into NHIS when they are involved in the programme. Previous studies have reported that where a critical analysis of various methods of identifying poor households is carried out, the community criteria of classifying the poorest members correlated with means testing and the proxy mean testing considered as the gold standard [26, 27].

Our study further shows that participants acknowledged the importance of the NHIS in increasing access to health care, but cited cost of treatment as the main reason that affected participants’ decision to get enrolled in the NHIS. Participants further mentioned poor quality of care, out-of pocket payment, far travel distance to NHIS registration/renewal centre and delays in the process, discrimination and negative attitude of health workers towards insured clients as barriers to low NHIS enrolment. Our findings are consistent with earlier studies reporting factors, such as lengthy waiting times at registration centres, occasional shortage of registration materials and perceived poor quality of healthcare services, provider attitudes and peer pressure as major barriers to enrolment [28–30]. In 2019, the NHIS introduced the use of mobile phone renewal strategy to address some of the challenges related to waiting time and travel distance affecting the scheme and to further digitize enrolment in the near future [31].

Although the study was conducted in three geographical regions representing the three main ecological zones of Ghana (southern, middle and northern zones), there were limitations. It must be noted that as a qualitative study, the findings from this study implementation context and dynamics are only applicable to other contexts of similar characteristics. The political context influencing selection of indigents in this study may not be applicable in other settings.

## Conclusions

There is a general belief that the LEAP process has been politicized and therefore favours only people who are noted to be sympathizers of the ruling government. Participants are also of the view that the process of selecting beneficiaries lacks transparency. The involvement of community leaders such as chiefs, opinion leaders and religious leaders could improve identification of the poor. There is therefore the need for stakeholders such as NHIA (National Health Insurance Authority), implementors of the LEAP, other civil society organizations, and religious leaders to engage the community to strengthen the approach to identifying and enrolling right households and individuals as indigents through the LEAP. Community-based approaches in the selection of the indigent are recommended to strengthen the NHIS-LEAP beneficiary process.

## Data Availability

The data is part of an ongoing study. In view of that we are restricted from sharing the large data set. However, the data contained in this manuscript are available upon a reasonable request to the corresponding author.

## Abbreviations

CHPS: Community-based Health Planning Services
DFID: Department for International Development
FGD: Focus Group Discussion
GSS: Ghana statistical services
IDI: In-Depth Interview
LEAP: Livelihood Empowerment against Poverty
NGO: Non-Governmental Organization
NHIA: National Health Insurance Authority
NHIS: National Health Insurance Scheme
OVC: Orphan or vulnerable children

## References

[1] Morestin F, Grant P, Ridde V. Criteria and processes for identifying the poor as beneficiaries of programs in developing countries. Univ Montréal. 2009.

[2] World Bank. World development report 2000/2001: attacking poverty. World Dev Report; New York Oxford Univ Press. 2001.

[3] Wagstaff A. Research on equity, poverty and health outcomes: lessons for the developing world. HNP Discuss Pap Ser World Bank, Washington, DC © World Bank [Internet]. 2000. https://openknowledge.worldbank.org/handle/10986/13680. License: CC BY 3.0 IGO.

[4] International Labour Office. Social protection global policy trends 2010–2015—from fiscal consolidation to expanding social protection: key to crisis recovery, inclusive development and social justice. Int Labour Off - Geneva ILO. 2014.

[5] Handa S, Park M, Darko R, Osei-Akoto I, Davis B, Diadone S. Livelihood Empowerment against Poverty Program impact evaluation. Carolina Popul Cent Univ North Carolina Chapel Hill. 2014.

[6] Okoroh J, Essoun S, Seddoh A, Harris H, Weissman JS, Dsane-Selby L (2018). Evaluating the impact of the national health insurance scheme of Ghana on out of pocket expenditures: a systematic review. BMC Health Serv Res.

[7] National Health Insurance Authority. National Health Insurance ACT (ACT 852). 2012. http://asgmresearch.weebly.com/uploads/3/0/1/6/30160743/national-health-insurance-act-2012-act-852.pdf.

[8] Foli R (2015). Transnational actors and policymaking in Ghana: the case of the Livelihood Empowerment against Poverty. Glob Soc Policy.

[9] National Development Planning Commission. Ghana millennium development goals: 2015 reports. Natl Dev Plan Comm Ghana. 2015.

[10] Ghana Statistical Service. Ghana poverty mapping report. Ghana Stat Serv. 2015.

[11] Hanson K, Worrall E, Wiseman V. Targeting services towards the poor: a review of targeting mechanisms and their effectiveness. In: Health, economic development and household poverty from understanding to action. Routledge. 2007.

[12] Ghana Statistical Service. 2010 population and housing census: final results. Ghana Stat Serv Final results. 2012;11.

[13] Ghana Statistical Services. Ghana living standards survey round 7. Ghana Stat Serv. 2018.

[14] Ofori-Birikorang A. Promoting a new health policy in the Ghanaian media: newspaper framing of the National Health Insurance Scheme from 2005–2007. A Diss Present to Fac Scripps Coll Commun Ohio Univ Partial fulfillment Requir degree Dr Philos. 2009.

[15] Ritchie J, Lewis J, Nicholls CM, Ormston R. Qualitative research practice: a guide for social science students and researchers. SPE Proc Gas Technol Symp. 2013.

[16] deMarrais KB, Lapan SD (Eds. ). Foundations for research: methods of inquiry in education and the social sciences (1st ed.). Routledge. 2003;51–68. https://www.taylorfrancis.com/chapters/edit/10.4324/9781410609373-8/qualitative-interview-studies-learning-experience.

[17] Poland B (1995). Transcription quality as an aspect of rigor in qualitative research. Qual Inq.

[18] Creswell J (2013). Qualitative inquiry and research design.

[19] Farrington J, Harvey P, Slater R. Cash transfers in the context of pro—poor growth C. Discuss Pap OECD/DAC Povnet Risk Vulnerability Task Gr 1. 2005.

[20] Tesliuc ED, Milazzo A. Reducing error, fraud and corruption (EFC) in social protection programs. 2013;1–6.

[21] Agbenyo F, Galaa SZ, Abiiro GA (2017). Challenges of the targeting approach to social protection: an assessment of the Ghana livelihood empowerment against poverty programme in the Wa Municipality of Ghana. Ghana J Dev Stud.

[22] Atulley KA. An assessment of the Livelihood Empowerment against Poverty Programme in the Bongo District, Ghana. A Thesis Submitt to Sch Grad Stud Kwame Nkrumah Univ Sci Technol Partial fulfillment Requir degree Master Sci Dev Policy Plan Dep Plan Coll Arc [Internet]. 2015. http://ir.knust.edu.gh/xmlui/bitstream/handle/123456789/9480/KENNEDY ATINGA ATULLEY.pdf?sequence=1.

[23] Kotoh AM, Van Der Geest S (2016). Why are the poor less covered in Ghana’s national health insurance? A critical analysis of policy and practice. Int J Equity Health.

[24] Dixon J, Luginaah I, Mkandawire P (2014). The National Health Insurance Scheme in Ghana’s upper west region: a gendered perspective of insurance acquisition in a resource-poor setting. Soc Sci Med.

[25] Ir P, Decoster K, Hardeman W, Horemans D, Damme WV (2008). An assessment of household eligibility for Health Equity Fund after four years of pre-identification in Oddar Meanchey, Cambodia. Hso&P.

[26] Aryeetey GC, Jehu-Appiah C, Spaan E, D’Exelle B, Agyepong I, Baltussen R (2010). Identification of poor households for premium exemptions in Ghana’s National Health Insurance Scheme: empirical analysis of three strategies. Trop Med Int Health.

[27] Atinga R, Abotisem GA, Kuganab-Lem R (2015). Factors influencing the decision to drop out of health insurance enrolment among urban slum dwellers in Ghana. Trop Med Int Health.

[28] Dror DM, Hossain SAS, Majumdar A, Pérez Koehlmoos TL, John D, Panda P (2016). What factors affect voluntary uptake of community-based health insurance schemes in low- and middle-income countries? A systematic review and meta-analysis. PLoS ONE.

[29] Fenny AP, Yates R, Thompson R (2018). Social health insurance schemes in Africa leave out the poor. Int Health.

[30] Jehu-Appiah C, Aryeetey G, Agyepong I, Spaan E, Baltussen R (2012). Household perceptions and their implications for enrolment in the National Health Insurance Scheme in Ghana. Health Policy Plan.

[31] Boaheng JM, Amporfu E, Ansong D, Osei-fosu AK (2019). Determinants of paying national health insurance premium with mobile phone in Ghana: a cross-sectional prospective study. Int J Equity Health.

